# Determinants of misuse of antibiotics among parents of children attending clinics in regional referral hospitals in Tanzania

**DOI:** 10.1038/s41598-022-08895-6

**Published:** 2022-03-22

**Authors:** Ritah F. Mutagonda, Alphonce I. Marealle, Lilian Nkinda, Upendo Kibwana, Betty A. Maganda, Belinda J. Njiro, Harrieth P. Ndumwa, Manase Kilonzi, Wigilya P. Mikomangwa, Hamu J. Mlyuka, Fatuma F. Felix, David T. Myemba, Dorkasi L. Mwakawanga, Godfrey Sambayi, Peter P. Kunambi, Pacifique Ndayishimiye, Nathanael Sirili, Rashid Mfaume, Arapha Nshau, Elevanie Nyankesha, Robert Scherpbier, George M. Bwire

**Affiliations:** 1 School of Pharmacy, Muhimbili University of Health and Allied Sciences, P.O. Box 65001, Dar es Salaam, Tanzania; 2 School of Medicine, Muhimbili University of Health and Allied Sciences, P.O. Box 65001, Dar es Salaam, Tanzania; 3 School of Nursing, Muhimbili University of Health and Allied Sciences, P.O. Box 65001, Dar es Salaam, Tanzania; 4 Department of Pharmacy, University of Rwanda, P.O. Box 4285, Kigali, Rwanda; 5 School of Public Health and Social Sciences, Muhimbili University of Health and Allied Sciences, P.O. Box 65001, Dar es Salaam, Tanzania; 6 Regional Administrative Secretary, Dar Es Salaam Region, P.O. Box 5429, Dar es Salaam, Tanzania; 7 Pharmacy Council, Ministry of Health, Community Development, Gender, Elderly and Children, P.O. Box 31818, Dar es Salaam, Tanzania; 8 United Nations Children’s Fund, New York Head Quarter, 3 United Nations Plaza, New York, NY 10017 USA; 9 United Nations Children’s Fund, Bâtiment BIT, Route des Morillons 4, 1211 Geneva 22, Switzerland

**Keywords:** Immunology, Microbiology, Diseases, Health care, Medical research

## Abstract

Parents are the important implementers on appropriate/inappropriate use of antibiotics, especially in the pediatric population. Limited studies have associated poor knowledge, attitude, and practice (KAP) among parents with antibiotics misuse. Therefore, this study was conducted to determine the parents’ KAP and factors associated with inappropriate use of antibiotics among Tanzanian children. A hospital-based cross-sectional study was conducted in 14 regional referral hospitals (RRHs) in Tanzania between June and September 2020. KAP was estimated using a Likert scale, whereas KAP factors were determined using logistic regression models. A total of 2802 parents were enrolled in the study. The median age (interquartile range) of parents was 30.0 (25–36) years where 82.4% (n = 2305) were female parents. The majority of the parents had primary education, 56.1% (n = 1567). Of 2802 parents, only 10.9% (n = 298) had good knowledge about antibiotics, 16.4% (n = 455) had positive attitude whereas 82.0% (n = 2275) had poor practice on the appropriate use of antibiotics. Parents' education level, i.e., having a university degree (aOR: 3.27 95% CI 1.62–6.63, p = 0.001), good knowledge (aOR: 1.70, 95% CI 1.19–2.23, p = 0.003) and positive attitudes (aOR: 5.56, 95% CI 4.09–7.56, p < 0.001) were significantly associated with the appropriate use of antibiotics in children. Most parents had poor knowledge, negative attitude, and poor practice towards antibiotics use in children. Parents’ education level, employment status, knowledge on antibiotic use, and good attitude contributed to the appropriate use of antibiotics in children attending clinics at RRHs.

## Introduction

Antibiotic misuse contributed by either antibiotic abuse, overuse or sub-optimal use has potential serious effects on health including development of antibiotic resistance. Antibiotics resistance (AR) is an inherent nature of the bacteria as a means of survival^1^. The impacts of AR are immense and broad; for instance, AR can result into prolonged hospital stay, financial burden, morbidity and mortality^2^. In 2019 World Health Organization (WHO) report estimated that AR resulted in around 700,000 annual deaths worldwide^3^ and by 2030 AR is estimated to contribute 10 million deaths worldwide^4^. The situation in low and middle-income countries (LMCIs), including Tanzania, is expected to be worse due to poverty, poor health care system, inadequate water supply and sanitation, and high human-animal interaction, which facilitate the emergence and spread of resistant strains^5^.

Several measures, such as rational use of antibiotics, need to be implemented to mitigate resistant bacteria^2^. Sub-optimal exposure to antibiotics due to poor adherence, underdose, and wrong indications elicits selection pressure and the emergence of resistant strains^6^. In recognition of this global public health threat, surveillance and dedicated implementational research may enable timely prevention and control of this emerging problem^7^. The strategies have to consider a holistic approach taking into account the key stakeholders including policy makers, scientists, donors/funders and implementers such as healthcare providers and community, including parents/guardians^8^. At implementation levels, some of the strategies include AR stewardship programs that promote antibiotics' rational use, antibiotic use evaluation and monitoring in health facilities, healthcare providers' training, creating community awareness, and one health initiative^9^.

On the other hand, when a patient, especially a child (outpatient), exit the hospital settings, instructions from the attending physician on rational antibiotic use need to be implemented by his/her parents^8^. In this note, parents or guardians are proxy to appropriate/inappropriate use of antibiotics, especially in the pediatric population^10^. In several studies, antibiotics misuse has been associated with poor knowledge, attitude, and practice (KAP)^11–13^. Parents have been reported to provide children with antibiotics for symptomatic treatment of common cold, cough, fever, or respiratory diseases^14^. Therefore, a study was conducted to determine the parents/guardians’ KAP and factors associated with inappropriate use of antibiotics among Tanzanian children.

## Methods

### Study design and population

A hospital-based cross-sectional study was conducted to determine the level of KAP and factors associated with the inappropriate use of antibiotics in pediatric patients among parents at 14 regional referral hospitals (RRHs) in Tanzania Mainland; Temeke, Tumbi, Ruvuma, Lindi, Geita, Sekoutoure, Dodoma, Singida, Njombe, Sumbawanga, Iringa, Songwe and Arusha. These hospitals have beds capacity between 300 and 500^15^. This study was conducted between June and September 2020. Study areas were categorized into seven administrative zones of Tanzania mainland. Within each zone, two RRHs were selected to represent each zone. The selection of hospitals within each zone was based on community economic activities^15^ such as business, manufacturing, tourism, livestock keeping, farming and mining. Economic activities were associated with the inappropriate use of antibiotics in community settings^16^. Additionally, RRHs were chosen as study sites because, according to the national action plan on antimicrobial resistance^8^, RRHs are listed as one of the implementers of the plan. Tanzania RRHs offer a range of clinical services such as pediatrics, outpatient and inpatient, emergency services, diagnostic (laboratory, radiology and imaging), pharmaceutical supply, internal medicine, surgery, obstetrics and gynecology^17^. The study population constituted of parents/guardians attending clinic at the respective RRHs.

### Sample size and sampling techniques

The sample size of parents/guardians interviewed was calculated using the formula; n = Z^2^*P (1 − P)/d^2^. Where n was the sample size, p (47.7%) was prevalence from the study conducted among Tanzanian parents^18^, (1 − α) = 0.95, Z_(1-α)_ = 1.96 for 95% confidence level, and the absolute error/precision (d) was assumed to be 0.022. A minimum sample size of 730 was required as previously described by Simon and Kazaura^18^.

Since the RRHs in Tanzania attend both primary and referred patients^17^, the consecutive sampling technique was used when enrolling parents or guardians so that both primary and referred patients were evenly selected. Each hospital contributed almost an equal number of participants.

### Data collection

#### Development and validation of a questionnaire.

The questionnaire was developed after a comprehensive literature review of the studies highlighting the influence of KAP on the irrational use of antibiotics in pediatric patients^19–22^. Some of the questions were adopted^23^ while other questions were customized to suit parents (assuming that most of the parents were non-medical professionals). The questionnaire was prepared in English and translated to Kiswahili language for convenient and accurate data collection. Ten individuals with a firm command of both languages checked the accuracy and meaning of the translated content. The questionnaire was first validated and tested to answer the study's objectives by a random selection of 10 pediatric parents in a pilot study conducted in Dar es Salaam region between 1 and 5 June 2020.

### Types of data collected

Data were collected using structured questionnaires. Each questionnaire consisted of five sections. Section A: collected data on socio-demographic information of the study participants (parents), which included age, gender, family type, number of children, education qualification, marital status, and residence, plus some socio-demographic information of the child such as age and sex. Section B: consisted of questions on knowledge about the use of antibiotics. Section C: comprised of questions on the influence of attitude on the use of antibiotics. Section D: consisted of questions assessing parental practice on the appropriate use of antibiotics. The grading used a Likert scale with choices from strongly disagree to strongly agree for knowledge and attitude, and always to never for practice. Section E consisted of questions on the parents’ sources of information about antibiotics (Additional file 1).

### Data analysis

Data were collected using Open Data Kit (ODK software, USA) and then exported to Microsoft Excel Sheet (Redmond, WA). Data coding and analysis were performed using Statistical Package for Social Sciences version 25 (SPSS Software, Chicago Inc., USA). Categorical variables such as sex, education level and marital status were presented using frequencies and percentages. Means and standard deviations were used in summarizing normal distributed variable while medians and interquartile ranges were used when variables were not normal distributed. A particular question was categorized as correct/uncertain/not correct for knowledge during data presentation, whereas yes/uncertain/no for attitude and yes/no for practice. Yes, was for strongly agree and agree responses while no was for strongly disagree, disagree, and uncertain for practice and attitude responses.

The overall knowledge, attitude and practice were estimated as previously described elsewhere^13,24^. Briefly, to every individual question, the correct response was given a score of 1, while incorrect response and uncertainty were given 0. Strongly agree and agree were merged as agree while strongly disagree and disagree merged as disagree. Depending on the score, knowledge was termed as poor, moderate, and good, while attitude was categorized as negative, uncertain, and positive. Poor/negative was given a score of 0–49%, moderate/uncertain 50–79% and good/positive 80–100%. Always and often were merged as agree, whereas sometimes/seldom/never were merged as disagree. For this research, questions on practice were used to measure appropriate/inappropriate use of antibiotics. The percentage score for practice was inappropriate (misuse) if the score was 0–79% and appropriate (good use) if the score was 80–100%.

The score was determined as the percentage of correct responses to the total asked questions in each category (K/A/P). An ordinal logistic regression model was used to determine factors associated with positive attitude and knowledge. The primary outcome of this study was the inappropriate use of antibiotics whereby, the binary logistic regression model was used to determine factors associated with misuse of antibiotics in children. The measure of associations was the crude odds ratio (cOR) for univariate analysis and adjusted odds ratio (aOR) for multivariate analysis. Multivariate analysis was done to all factors that showed statistics p < 0.2 in the univariate analysis. The p-value of less than 0.05 at the confidence interval of 95% (95%CI) indicated the statistical significance.

### Ethics approval and consent to participate

Ethical approval to conduct this study was obtained from Muhimbili University of Health and Allied Sciences Ethical Review Board (Reference number: MUHAS-REC-3-2020-109). Permission to conduct the study at the pediatric departments were requested from the Medical Officer In-charges. Written informed consents after explaining the purpose of the study were requested from parents before enrollment. Addition, this study was conducted with accordance to the law and regulation of Tanzania.

## Results

### Participants’ sociodemographic characteristics

A total of 2802 parents and 2793 children attending clinics in the selected RRHs in Tanzania were included in this study. The median age (interquartile range) of parents was 30.0 (25–36) years, while the median age for their children attending the clinic was 2.0 (1–4) years. About half of the parents (50.6%) were in the age between 25 and 35 years (n = 1408) whereas children with age less than 5 years were the majority 88.1% (n = 2460), and 82.4% (n = 2305) were female parents. About 81% (2259) were married whilst 56.1% (n = 1567) had attained primary education, 63.8% (n = 1782) lived in a nuclear family type and 45.7% (n = 1276) of the parents had more than 2 children. Almost half of the parents 46.8% (n = 1307) were self-employed and 51.2% (n = 1428) had low income. Most of the children were males 53.3% (n = 1484) whereas 68.3% (n = 1823) of their parents lived in urban areas (Table 1).

**Table 1:** Parents’ and children’s sociodemographic information.

Variable	Category	Frequency (n)	Percent (%)
Parent’s age (years)	< 25	621	22.3
	25–35	1408	50.6
	36–45	612	22.0
	> 45	142	5.1
Parent’s sex	Male	493	17.6
	Female	2305	82.4
Marital status	Married	2259	81.0
	Not married	530	19.0
Parent’s education level	Illiterate	271	9.7
	Literate	418	15.0
	Primary	1567	56.1
	Secondary	213	7.6
	Certificate	100	3.6
	Diploma	122	4.4
	Graduate	101	3.6
	Postgraduate	3	0.1
Employment status	Employed	576	20.6
	Self employed	1307	46.8
	Not employed	909	32.6
Family type	Nuclear family	1782	63.8
	Single parent family	511	18.3
	Extended family	499	17.9
Number of children	1	723	25.9
	2	794	28.4
	≥ 3	1276	45.7
Child’s sex	Male	1484	53.3
	Female	1300	46.7
Child’s age (years)	< 5	2460	88.1
	5–9	276	9.9
	≥ 10	57	2.0
Family monthly income^a^ ($)	Low	1428	51.2
	Middle	1133	40.6
	High	228	8.2
Place of residence	Urban	1823	68.3
	Rural	847	31.7

^a^The classification of family income used a world bank definition of poverty of population living on less than $1.25 per day.

### Responses from parents on the individual questions

**Figure 1:**
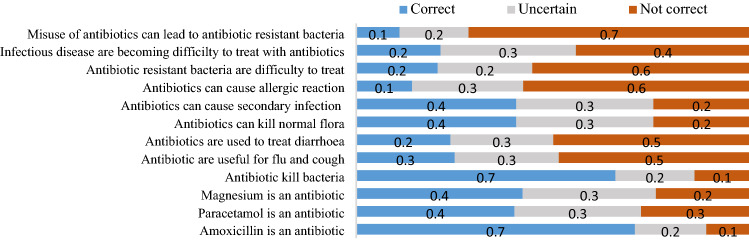
Knowledge on rational use of antibiotics in children among parents attending clinic at RRHs in Tanzania. In assessing the knowledge, 2726 participants responded to different questions whereby 42.3% and 40.1% knew that magnesium and paracetamol are not antibiotics, respectively. Few participants, 11% had the knowledge that misuse of antibiotics can lead to AR while 21.4% had adequate knowledge on AR.

**Figure 2:**
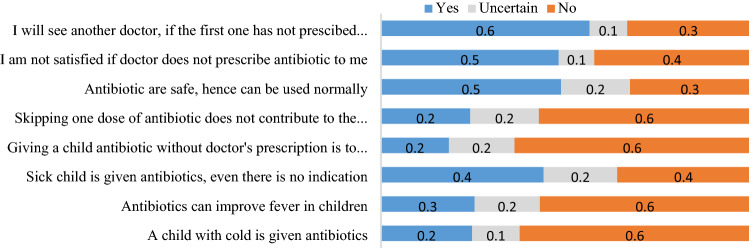
Parents’ attitude toward rational use of antibiotics among children. Among 2772 parents assessed for their attitude towards appropriate antibiotic use in children, 56.0% had a negative attitude to non-antibiotics prescriptions and were ready to seek another doctor who would prescribe an antibiotic for their children. The majority (63.8%) were ready to give their children antibiotics without a doctor’s prescription. Additionally, 44.1% could provide antibiotics for their children, even without an indication.

**Figure 3:**
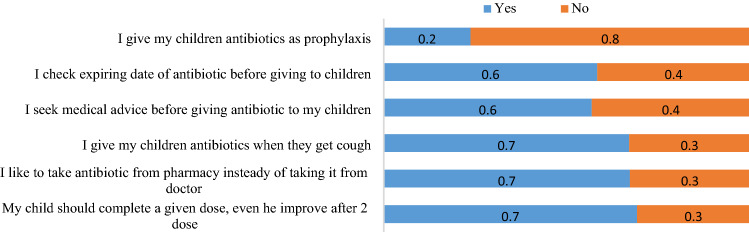
Parental practice on the appropriate use of antibiotics in children. Of 2775 responded to the practice questions, 67.0% like to take medicine from pharmacies than doctors. Some parents (30.3%) were ready to stop giving antibiotics to their children when there were improvements. About 33% of parents gave antibiotics to their children when they had a cough. Regarding the expiry date, 41.7% reported that they don’t check the expiring dates of antibiotics before giving to their children.

Figures 1, 2 and 3 highlight the responses from parents on knowledge, attitude and practice against the appropriate use of antibiotics for children.

### Parents’ sources of information about antibiotics

Majority of the parents obtained their information from dispensers 85.6% (n = 2385) followed by prescriber 77.1% (n = 2148) then nurses 76.1% (n = 2131). Few parents obtained the information about antibiotics from social media 26.0% (n = 724), colleague 25.5% (n = 706) and university courses 15.6% (n = 434) (Table 2).

**Table 2:** Parents’ source(s) of information about antibiotics.

Source	Frequency (n)	Percent (%)
Information provided by pharmaceutical companies leaflet	1280	45.7
Information from prescribers	2148	77.1
Information from dispensers	2385	85.6
Information from nurses	2131	76.1
Information given by a colleague	706	25.5
Information from University courses	434	15.6
Internet	831	29.8
Social media	724	26.0
Others	196	7.0

A parent was able to choose more than one source. Hence, the total percentage is more than 100%.

### Source(s) of information about antibiotics (Fig. 4)

**Figure 4:**
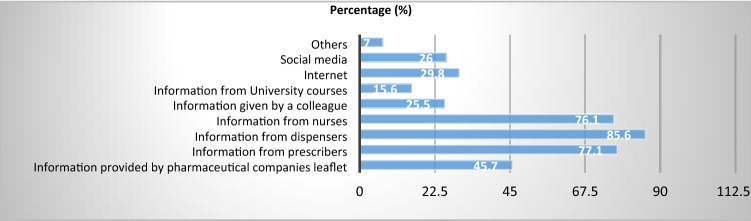
Parents’ source(s) of information about antibiotics. Of 2802 respondents, majority of the parents obtained their information from dispensers 85.6% followed by prescriber 77.1%) then nurses 76.1%. Few parents obtained the information about antibiotics from social media 26.0%, colleague 25.5% and university courses 15.6%.

#### Overall parents’ KAP on rational use of antibiotics in children

Out of 2802 parents who were assessed on the KAP, only 10.9% (n = 298) had good knowledge, 16.4% (n = 455) had a positive attitude and 82.0% (n = 2275), had poor practice on rational antibiotics use in children (Table 3).

**Table 3:** Overall parents’ KAP on rational use of antibiotics in children.

Variable	Categories	Frequency (n)	Percentage (%)
Knowledge	Poor	1272	46.7
	Moderate	1156	42.4
	Good	298	10.9
Attitude	Negative	1485	53.6
	Uncertain	832	30.0
	Positive	455	16.4
Practices	Poor	2275	82.0
	Good	500	18.0

### Factors associated with knowledge of antibiotics use in children

Both univariate and multivariate analysis found an association between parents’ age and knowledge about antibiotics use in children. Parents with age between 36 and 45 years had 41% high odds (adjusted odds ratio [aOR]: 1.41, 95% CI 1.05–1.90, p = 0.021) of good knowledge about antibiotics use compared to those < 25 years. Employed parents had about 3 times more chance of having good knowledge than unemployed parents (aOR: 2.66, 95% CI 2.06–3.43, p < 0.001). Parents with a degree had good knowledge about antibiotics use in children compared to illiterate parents (aOR: 3.22, 95% CI 1.96–5.26, p < 0.001). Parents living in a nuclear family (father, mother, children) had poor knowledge when compared to those living in the extended family (aOR: 0.71, 95% CI 0.58–0.88, p = 0.002) while parents living in urban areas had 64% more chance to have good knowledge as compared to those from rural areas (aOR: 1.64, 95% CI 1.37–1.96, p < 0.001) (Table 4).

**Table 4:** Univariate and multivariate analysis of factors associated with parents’ good knowledge about antibiotics use in children.

Variable	Categories	Univariate analysis			Multivariate analysis		
		cOR	95% CI	P-value	aOR	95% CI	P-value
Parents’ age (years)	> 45	1.41	0.99–2.01	0.054	1.55	1.02–2.36	0.041
	36–45	1.60	1.28–1.99	< 0.001	1.41	1.05–1.90	0.021
	25–35	1.47	1.22–1.77	< 0.001	1.13	0.90–1.43	0.287
	< 25	Ref			Ref		
Number of children	≥ 3	1.12	0.94–1.34	0.197	0.80	0.62–1.03	0.079
	2	1.28	1.05–1.55	0.015	1.00	0.79–1.26	0.991
	1	Ref			Ref		
Parents’ sex	Female	0.96	0.80–1.16	0.698			
	Male	Ref					
Marital status	Married	1.46	1.21–1.76	< 0.001	1.15	0.86–1.54	0.351
	Not married	Ref			Ref		
Occupation	Employed	3.81	3.09–4.69	< 0.001	2.66	2.06–3.43	< 0.001
	Self-employed	1.33	1.13–1.57	0.001	1.21	1.002–1.45	0.048
	Not employed	Ref			Ref		
Education level	Degree/Masters	7.58	4.83–11.91	< 0.001	3.22	1.96–5.26	< 0.001
	Diploma	3.90	2.56–5.93	< 0.001	1.40	0.87–2.24	0.167
	Certificate	6.42	4.10–10.07	< 0.001	2.87	1.76–4.66	< 0.001
	Advanced secondary	1.20	0.84–1.73	0.317	0.68	0.46–1.02	0.065
	Primary/secondary	2.01	1.55–2.62	< 0.001	1.41	1.06–1.88	0.018
	Literate	1.25	0.92–1.70	0.159	0.85	0.61–1.19	0.350
	Illiterate	Ref			Ref		
Family type	Nuclear family	0.86	0.71–1.04	0.133	0.71	0.58–0.88	0.002
	Single parent family	0.58	0.46–0.74	< 0.001	0.61	0.44–0.84	0.003
	Extended family	Ref			Ref		
Place of residence	Urban	2.06	1.75–2.45	< 0.001	1.64	1.37–1.96	< 0.001
	Rural	Ref			Ref		

*cOR* crude odds ratio, *aOR* adjusted odds ratio, *95%CI* 95% confidence interval, *Ref* reference category.

### Factors associated with attitude on appropriate use of antibiotics in children

Univariate analysis found an association between positive attitude and parents’ age whereby parents aging between 25 and 45 years had 35% more chance to have a positive attitude as compared to those with age below 25 years (cOR: 1.35, 95% 95% CI 1.08–1.68, p = 0.07). However, there was no association on multivariate analysis (aOR: 1.16. 95% CI 0.91–1.49, p = 0.234). Female parents had negative attitude on antibiotics use in children compared to male parents (aOR: 0.76, 95%: 0.62–0.94, p = 0.01). Degree holder parents had five times more chance to have a positive attitude towards antibiotics use in children than illiterate parents (aOR: 5.3, 95% CI 3.24–8.65, p < 0.001). Patients living in urban areas were 2 times likely to have a positive attitude as compared to those from rural areas (aOR: 2.07, 95% CI 1.72–2.49, p < 0.001) (Table 5).

**Table 5:** Univariate and multivariate analysis of factors associated with parents’ positive attitude on appropriate use of antibiotic in children.

Variable	Categories	Univariate analysis			Multivariate analysis		
		cOR	95% CI	P-value	aOR	95% CI	P-value
Parents’ age (years)	> 45	1.08	0.76–1.54	0.677	1.19	0.79–1.77	0.405
	36–45	1.35	1.08–1.68	0.007	1.16	0.91–1.49	0.234
	25–35	1.33	1.10–1.60	0.003	1.10	0.90–1.35	0.357
	< 25	Ref			Ref		
Number of children	≥ 3	1.07	0.90–1.28	0.447			
	2	1.20	0.99–1.46	0.063			
	1	Ref					
Parents’ sex	Female	0.81	0.68–0.98	0.029	0.76	0.62–0.94	0.010
	Male	Ref			Ref		
Marital status	Married	1.28	1.06–1.54	0.010	1.03	0.77–1.38	0.844
	Not married	Ref			Ref		
Occupation	Employed	1.93	1.58–2.36	< 0.001	1.20	0.94–1.55	0.148
	Self-employed	1.19	1.01–1.40	0.040	0.96	0.80–1.15	0.634
	Not employed	Ref			Ref		
Education level	Degree/masters	8.79	5.60–13.82	< 0.001	5.30	3.24–8.65	< 0.001
	Diploma	2.54	1.66–3.89	< 0.001	1.38	0.85–2.23	0.187
	Certificate	4.92	3.15–7.70	< 0.001	2.89	1.77–4.70	< 0.001
	Advanced secondary	1.16	0.79–1.71	0.441	0.76	0.49–1.17	0.214
	Primary/secondary	2.73	2.06–3.60	< 0.001	1.92	1.42–2.60	< 0.001
	Literate	1.32	0.95–1.84	0.097	0.92	0.65–1.32	0.661
	Illiterate	Ref			Ref		
Family type	Nuclear family	1.22	1.004–1.48	0.046	1.08	0.87–1.33	0.493
	Single parent family	0.81	0.64–1.04	0.093	0.81	0.59–1.13	0.224
	Extended family	Ref			Ref		
Residence	Urban	2.37	2.00–2.81	< 0.001	2.07	1.72–2.49	< 0.001
	Rural	Ref			Ref		

*cOR* crude odds ratio, *aOR* adjusted odds ratio, *95%CI* 95% confidence interval, *Ref* reference category.

### Influence of sociodemographic, knowledge and attitude on the appropriate use of antibiotics in children

Factors for appropriate use of antibiotics in children were assessed. The univariate analysis found that employed parents were 3 times more likely to have good practice as compared to un-employed parents (cOR: 2.79, 95% CI 2.13–3.64, p < 0.001), but the multivariate analysis found no association (aOR: 1.18, 95% CI 0.78–1 78, p = 0.431). Parents' education significantly influenced the appropriate use of antibiotics in children, i.e., patients with at least a bachelor's degree had 3 times more chance of having good practice than non-educated parents (aOR: 3.27 95% CI 1.62–6.63, p = 0.001). Parents living in a nuclear family (father, mother and children) had 35% more chance of having a good practice compared to those from an extended family (father, mother, children and relatives) (cOR: 1.35, 95% CI 1.03–1.77, p = 0.032) while being an urban resident had 76% more chance of giving antibiotics to a child correctly (cOR: 1.76, 95% CI 1.39–2.23, p < 0.001). Regarding the knowledge and attitude toward the good practice of antibiotics use in children. Parents with a good knowledge had 70% more chance of having good practice (aOR: 1.70, 95% CI 1.19–2.23, p = 0.003); similarly, those with positive attitudes had about 6 times more chance (aOR: 5.56, 95% CI 4.09–7.56, p < 0.001) of practicing appropriately (Table 6).

**Table 6:** Factors associated with appropriate use of antibiotics in children (good practice).

Variable	Categories	Univariate analysis			Multivariate analysis		
		cOR	95% CI	P-value	aOR	95% CI	P-value
Parents’ age (years)	> 45	0.58	0.32–1.06	0.074	0.82	0.40–1.65	0.573
	36–45	1.04	0.77–1.42	0.788	0.99	0.64–1.55	0.994
	25–35	1.37	1.07–1.77	0.014	1.05	0.76–1.46	0.769
	< 25	Ref			Ref		
Children’s (years)	≥ 10	0.75	0.35–1.60	0.459			
	5–9	0.80	0.56–1.12	0.193			
	< 5	Ref					
Number of children	≥ 3	0.70	0.55–0.88	0.003	0.66	0.46–0.94	0.023
	2	0.94	0.73–1.21	0.627	0.82	0.59–1.12	0.212
	1	Ref			Ref		
Parents’ sex	Female	1.38	1.05–1.82	0.020	1.51	1.08–2.09	0.015
	Male	Ref			Ref		
Marital status	Married	1.13	0.87–1.45	0.363			
	Not married	Ref					
Occupation	Employed	2.79	2.13–3.64	< 0.001	1.18	0.78–1.78	0.431
	Self-employed	1.44	1.13–1.84	0.003	1.16	0.85–1.57	0.348
	Not employed	Ref			Ref		
Monthly income^a^ ($)	High	4.52	3.29–6.20	< 0.001	2.02	1.29–3.16	0.002
	Middle	2.35	1.90–2.92	< 0.001	1.53	1.14–2.04	0.004
	Low	Ref					
Education level	Degree/Masters	11.13	6.16–20.12	< 0.001	3.27	1.62–6.63	0.001
	Diploma	4.94	2.74–8.93	< 0.001	2.10	1.05–4.22	0.036
	Certificate	5.06	2.73–9.39	< 0.001	1.67	0.83–3.37	0.151
	Advanced secondary	2.17	1.21–3.87	0.009	1.10	0.56–2.14	0.791
	Primary/secondary	2.83	1.78–4.49	< 0.001	1.46	0.88–2.41	0.145
	Literate	0.95	0.53–1.69	0.864	0.64	0.34–1.20	0.162
	Illiterate	Ref			Ref		
Family type	Nuclear family	1.35	1.03–1.77	0.032	1.19	0.86–1.65	0.296
	Single parent family	1.06	0.76–1.50	0.729	1.03	0.67–1.58	0.905
	Extended family	Ref			Ref		
Place of residence	Urban	1.76	1.39–2.23	< 0.001	0.82	0.62–1.09	0.170
	Rural	Ref			Ref		
Knowledge	Good	4.44	3.31–5.95	< 0.001	1.70	1.19–2.43	0.003
	Moderate	1.85	1.47–2.31	< 0.001	1.23	0.95–1.59	0.112
	Poor	Ref			Ref		
Attitude	Positive	7.51	5.78–9.77	< 0.001	5.56	4.09–7.56	< 0.001
	Uncertain	3.28	2.57–4.19	< 0.001	2.80	2.12–3.70	< 0.001
	Negative	Ref			Ref		

^a^The classification of family income used a world bank definition of poverty of population living on less than $1.25 per day.

## Discussion

Due to many infectious diseases in developing countries like Tanzania, antibiotics use in children is widespread. This study surveyed 2802 parents KAP towards antibiotic use in children. To our knowledge, this is the first national-wide study to assess parents' KAP in Tanzania regarding the appropriate use of antibiotics in children. The evidence-based data provided from this study can be used to properly design the national interventional programs to improve children's general use of antibiotics.

This study explored different knowledge domains related to the identification of antibiotics, antibiotics' role, side effects of antibiotics, and antibiotic resistance. On average, the study found that only 10% of the parents had good knowledge of antibiotics. The reported proportion is extremely low compared to the recent findings (62.7%) reported in an urban area in Moshi, Northern Tanzania^25^. The difference observed is not surprising since the study conducted in Moshi consisted of a small sample size of participants residing in an urban area, with only 1.7% of participants not having formal education. In knowledge specific questions, the majority (65.9%) of the parents knew that antibiotics are used for bacterial infection. However, most of them were either uncertain or did not know that viruses could cause some types of upper respiratory infections and diarrhea in children. Therefore, in this case, antibiotics are not sufficient. The proportion reported in this study was higher than what was previously reported in India. Only 28% knew that the antibiotics are used for bacterial infections; the rest were confused about using antibiotics for bacteria or viruses^26^.

One of the critical problems of inappropriate use of antibiotics is the development of antibiotic resistance. Regarding parents' knowledge of antibiotic resistance issues in this study, only 11% of the patients knew that misuse of antibiotics could lead to antibiotic-resistant bacteria. About 20.7% of the parents knew that antibiotic-resistant bacteria are difficult to treat. The observed proportion is lower than what was reported in studies conducted in relatively more developed countries such as; in Greece 88%, Cyprus 90%, Israel 78% and Japan 43% of parents were aware that antibiotic misuse might cause antibiotic resistance^27–30^. The correct description of antibiotics was also assessed in this study, whereby 65.9% of the parents could recognize amoxicillin as an antibiotic. More than 50% of the parents didn't know or were uncertain that paracetamol and magnesium trisilicate are not antibiotics suggesting poor parents' knowledge of antibiotics identification. This observation is similar to what was previously reported in Moshi urban, Tanzania, and rural China, whereby some of the study participants could not correctly tell ampicillin is an antibiotic^25,31^.

Several factors associated with good parental knowledge about antibiotics use in children have been identified in this study. One is formal education, which was strongly associated with good knowledge about antibiotic use in children. The findings correlate with a previous study conducted in India and a review report. Out of 10 studies that examined the association between education and knowledge of antibiotic use, nine found a significant association^14,26^. This study also found that parents with older age had good knowledge about antibiotics use in children than the younger parents. Similar findings have been reported elsewhere^28,32,33^. Parents’ occupation was strongly associated with good knowledge of antibiotics use in children, whereby employed parents were two times more likely to have good knowledge about antibiotic use in children than non-employed parents. However, a previous study conducted in a much smaller area in Moshi urban, Tanzania, indicated that occupation does not affect adults' knowledge of antibiotic use^25^. The discrepancy observed here could be because the previous study involved people in the urban area only.

Regarding parents' sources of knowledge about antibiotic use in children, most parents reported obtaining their information from dispensers (85.6%) and prescribers (77.1%). These findings suggest that most people mainly get knowledge once they visit a health facility, especially when they have health problems; thus, most people in the community are left without information. The use of other means of conveying knowledge like TV, radio and the internet need to be considered when planning intervention programs.

Parents attitude towards antibiotics use has significant implications for the irrational use of antibiotics, leading to the development of antibiotic resistance in children^34^. More than half (56%) had a negative attitude to a non-antibiotic's prescription in assessing parents' attitudes. They were ready to seek another doctor who would prescribe an antibiotic for their children. The proportion of parents demanding such inappropriate prescription for antibiotics reported in this study is almost twice as high as that reported in the previous study^35^. Parental pressure and expectation have instigated pediatricians to prescribe antibiotics more often than required to satisfy their clients^29,36^. It is thought that most of the antibiotic prescriptions in pediatrics are dispensed for the treatment of virus-related infections^37^.

Other parents (63.8%) were ready to give their children antibiotics without a doctor's prescription and 67% preferred to obtain their antibiotics at the community pharmacies. In most countries, including Tanzania, the community can access antibiotics through community pharmacies. The ease of obtaining antibiotics at the pharmacy was reported in a previous study in Moshi, Tanzania using a simulated client's approach whereby out of 82 pharmacies which were visited, 92.3% of retailers dispensed antibiotics without prescriptions^37^. Therefore, healthcare providers' willingness to dispense antibiotics without prescription has made it easy for the community to obtain the medication, increasing irrational antibiotic use. This observation highlights the pharmacists' vital role in educating the community/parents about the safe and effective use of antibiotics.

Additionally, 44.1% could provide antibiotics for their children, even without an indication, whereby more than 50% of all parents agreed they would give antibiotics for treatment of fever or cold or cough. Similarly, previous KAP studies in Malaysia and Palestine also reported that most parents believed that antibiotics were helpful in the treatment of fever^38,39^. The incorrect use of antibiotics has also been documented elsewhere, whereby antibiotics have been used in self-limiting infections, especially upper respiratory tract illnesses, which do not require antibiotic treatment^25,35^.

Good attitudes and acceptable practices on antibiotics use would result in antibiotics' rational use and reduce antibiotic resistance. This study demonstrates that parents with good knowledge had 70% more chance of having a good practice; similarly, those with positive attitudes had 6 times more chance of practicing appropriately.

Good knowledge and positive attitude have also affected the parents' practice on antibiotics use in other studies^25,35,40^. Besides knowledge and parents' attitude, other factors affecting good practice on antibiotics use were parents' education level. Parents with at least a bachelor's degree had 3 times more chances of having good practice than non-educated parents. The effect of education in the knowledge, attitude and the ultimate practice on antibiotics use has been observed in previous studies in developing and developed countries^25,41^. Living in a nuclear family was also associated with a 35% more chance of having a good practice than an extended family. The reason for this is not apparent, but we speculate that having a large number of people in a home might make it difficult for the parent to make sure the child has to take the prescribed dose of the antibiotic. It may also influence the parent to get the medicine from the nearest pharmacy without taking the child to the hospital.

This study was conducted among parents found at clinics in the selected RRHs in Tanzania. The type of hospitals may influence the responses from the parents. RRHs have adequate specialists and facilities such as laboratories and well-equipped pharmacies which could contribute to the reduction of antibiotics misuse unlike the health centers and dispensaries where sometimes the nurse attendant can prescribe and dispense medications without proper indication and adequate counselling to parents/guardians on rational use of antibiotics. RRHs are currently implementing national action plan on antimicrobial resistance^8^. Additionally, to minimize professional bias, this study only assessed out-patients. The reason to only include outpatients’ is because in-patients drug dispensing and utilization is done under the supervision of nurses. Enrollment of an almost equal number of participants at each study site and data analysis not accounting for the variations in the hospital bed capacity existing among the selected hospitals are among the limitations to this study. Lastly, the conclusion from this study is limited to parents of children attending clinics at RRHs.

In conclusion, most parents had poor knowledge, negative attitude, and poor practice (KAP) towards antibiotics use in children. Factors such as education level, employment status, knowledge and attitude were significantly associated with the appropriate use of antibiotics among parents of children attending clinics at RRHs. Education on appropriate use of antibiotics especially in children should be sustainably provided through radio, tv, magazine and. Community based study on antibiotics use in children from their parents is warranted.

## Supplementary Information

Supplementary Information.

## Data Availability

The datasets generated and/or analyzed during the current study are available from the corresponding author on reasonable request.

## Abbreviations

aOR: Adjusted odds ratio
AR: Antibiotics resistance
cOR: Crude odds ratio
KAP: Knowledge, attitude, practice
MUHAS: Muhimbili university of health and allied sciences
RRHs: Regional referral hospitals
UNICEF: United Nations Children’s Fund
