# Supplementary material for: Determinants of misuse of antibiotics among parents of children attending clinics in regional referral hospitals in Tanzania
Source: Sci Rep. 2022 Mar 22;12:4836. doi: 10.1038/s41598-022-08895-6 (PMC8941073; doi:10.1038/s41598-022-08895-6)
Supplement: Supplementary file 1 — Supplementary Information. [file 41598_2022_8895_MOESM1_ESM.docx]

**Determinants of Misuse of Antibiotics Among Parents of Children Attending Clinics in Regional Referral Hospitals in Tanzania**

Ritah Mutagonda^1^, Alphonce I. Marealle^1^, George M. Bwire^1^, Lilian Nkinda^2^, Upendo Kibwana^2^, Betty Maganda^1^, Manase Kilonzi^1^, Wigilya P. Mikomangwa^1^, Hamu J. Mlyuka^1^, Fatuma F. Felix^1^, David T. Myemba^1^, Dorkasi L. Mwakawanga^3^_,_ Godfrey Sambayi^1^, Peter P. Kunambi^2^, Pacifique Ndayishimiye^5*^_,_ Nathanael Sirili^4^

# APPENDIX 1C: KAP ON ANTIBIOTIC USE PEDIATRIC PATIENTS AMONG PARENTS

**Interview Date: __________________**

**Hospital name: __________________**

**SECTION A: Socio-Demographic Parent/Guardian**

| **No** | **Identification number of Respondent** | **Age (years)** | **Sex** | | **Education level** | **Marital status** |  |
| --- | --- | --- | --- | --- | --- | --- | --- |
| 01 |  |  |  | |  |  |  |
| **Note:**  **Sex:** 1. M 2. F  **Education:** 1. Illiterate, 2. Literate, 3. Primary/Secondary, 4. High School, 5. Intermediate, 6. Bachelors, 7. Masters, 8. PhD | | | | | | |  |
| 02 | What is your occupation (that is, what kind of work do you mainly do)? | | | 1. **Employed** 2. **Self-employed** 3. **Non-employed** | | | |
| 03 | What is your type of family? | | | Nuclear .………..……………………  Single …………….……………..…..  Extended………………………….… | | | |
| 04 | 1. How many children are in your family? 2. What is the age and sex of your sick child? | | | Total……………  Age: Days/Months/years……… | | | |
| 05 | What is your average monthly family income? | | | 1. < Tsh.150,000 2. Tsh. 150,000 – 1,000,000 3. > Tsh. 1,000,000 | | | |

**SECTION B: Knowledge of parents/guardians about antibiotics**

| **No** | **Questions** | **Coding Category** |
| --- | --- | --- |
| 06 | Have you ever heard of a type of medicine called antibiotics? | Yes………………1  No …..……………2 |

**Note:** If respondent says ‘No” please ask if they have heard of a widely used antibiotic such as penicillin or metronidazole before asking the questions from 07.

| **No** | **Domains** | **Questions** | **Response** | | | | |
| --- | --- | --- | --- | --- | --- | --- | --- |
|  |  |  | **Strongly Disagree** | **Disagree** | **Uncertain** | **Agree** | **Strongly Agree** |
| 07 | Identification of antibiotics | Amoxicillin is an antibiotic |  |  |  |  |  |
|  |  | Paracetamol is an antibiotic |  |  |  |  |  |
|  |  | Aluminium hydroxide+ Magnesium hydroxide (antacid) is an antibiotic |  |  |  |  |  |
| 08 | Knowledge on the role antibiotic | Antibiotics are useful for killing bacteria |  |  |  |  |  |
|  |  | Antibiotics are often needed for cold and flu illness |  |  |  |  |  |
|  |  | Diarrhoea gets better faster with antibiotics |  |  |  |  |  |
| 09 | Side-effects of antibiotics | Antibiotics can kill “good bacteria” present in our bodies |  |  |  |  |  |
|  |  | Antibiotics can cause secondary infections after killing good bacteria present in our bodies |  |  |  |  |  |
|  |  | Antibiotics can cause allergic reactions |  |  |  |  |  |
| 10 | Antibiotic resistance | If bacteria are resistant to antibiotics, it can be very difficult to treat the infections they cause |  |  |  |  |  |
|  |  | Many infections are becoming increasingly resistant to treatment by antibiotics |  |  |  |  |  |
|  |  | Misuse of antibiotics can lead to antibiotic resistance |  |  |  |  |  |

**SECTION C: Attitudes of parents/guardians on antibiotics use**

| **No.** | **Domains** | **Questions** | **Response** | | | | |
| --- | --- | --- | --- | --- | --- | --- | --- |
|  |  |  | **Strongly Disagree** | **Disagree** | **Uncertain** | **Agree** | **Strongly Agree** |
| 11 | Preference for use of antibiotics | When my child have a cold, I should give antibiotics to prevent getting a more serious illness. |  |  |  |  |  |
|  |  | When the child get a fever, antibiotics help the child to get better more quickly. |  |  |  |  |  |
|  |  | I would rather give an antibiotic to the child that may not be needed than wait to see if I get better without it. |  |  |  |  |  |
| 12 | Antibiotic resistance and safety | Whenever I give an antibiotic to the child, I contribute to the development of antibiotic resistance. |  |  |  |  |  |
|  |  | Skipping one or two doses does not contribute to the development of antibiotic resistance. |  |  |  |  |  |
|  |  | Antibiotics are safe drugs; hence they can be commonly used. |  |  |  |  |  |
| 13 | Attitudes to doctor’s prescribing of antibiotics | If I expect to receive an antibiotic for the child, I am less satisfied with a doctor’s visit if I do not receive an antibiotic. |  |  |  |  |  |
|  |  | If a doctor does not prescribe an antibiotic when I think one is needed, I will go to another doctor. |  |  |  |  |  |

# SECTION D: Practices of parents/guardians regarding antibiotics

| **No** | **Questions** | **Response** | | | | |
| --- | --- | --- | --- | --- | --- | --- |
|  |  | **Almost always** | **Often** | **Sometimes** | **Seldom** | **Never** |
| 14 | If your child feels better, after taking 2–3 doses of antibiotics, do you still administer complete full course of treatment? |  |  |  |  |  |
| 15 | Do you prefer to obtain antibiotics from the pharmacy rather than doctor/health worker if your child have an illness |  |  |  |  |  |
| 16 | Do you prefer to give an antibiotic when your child or children have cough or sore throat? |  |  |  |  |  |
| 17 | Do you consult a doctor before giving an antibiotic to your child? |  |  |  |  |  |
| 18 | Do you check the expiry date of the antibiotic before giving to your child it? |  |  |  |  |  |
| 19 | Do you provide antibiotics to your child (ren) as a prophylaxis |  |  |  |  |  |

**SECTION E: Sources of information about antibiotics**

| 1. What is your source(s) of information about antibiotics? (Tick all that apply) | |
| --- | --- |
| 1. Information provided by pharmaceutical companies leaflet |  |
| 1. Information from prescribers |  |
| 1. Information from dispensers |  |
| 1. Information from nurses |  |
| 1. Information given by a colleague |  |
| 1. Information from University courses |  |
| 1. Internet |  |
| 1. Antibiotic guidelines |  |
| 1. The World Health Organization’s (WHO) guidelines for treatment of bacterial diseases |  |
| 1. Social media |  |
| 1. Others | ……………….. |
